# Heat Illness and Deaths — New York City, 2000–2011

**Published:** 2013-08-09

**Authors:** Katherine Wheeler, Kathryn Lane, Sarah Walters, Thomas Matte

**Affiliations:** New York City Dept of Health and Mental Hygiene

Heat waves kill more persons, on average, than any other extreme weather event in the United States (1), and additional heat-related deaths are caused by hot weather not classified as heat waves (2). Summer temperatures in New York City (NYC) are increasing, with longer and hotter heat waves projected into the next century and beyond (3). To assess current risk factors and vulnerable populations among NYC residents, hospital data, death certificate data, and medical examiner records involving cases of heat illness, including hyperthermia (also known as heat stroke), were analyzed by the NYC Department of Health and Mental Hygiene for the period 2000–2011. On average, 447 patients each year were treated for heat illness and released from emergency departments, 152 were hospitalized, and 13 persons died from heat stroke. Chronic diseases, mental health disorders, and obesity were common comorbidities. Among fatality investigation records with information available about cooling, none found a working air conditioner in use. Outreach to city residents at high risk and their caregivers should emphasize the dangers of heat and importance of protective cooling measures during hot weather. Improving awareness of chronic health conditions that increase vulnerability to heat is also important.

The New York Statewide Planning and Research Cooperative System (SPARCS) provided de-identified electronic patient records from NYC emergency departments (EDs) and hospitals from 2000 through 2010.* Records were limited to NYC residents and homeless persons with any diagnosis of heat illness from *International Classification of Diseases, Ninth Revision, Clinical Modification* (ICD-9-CM) codes 992.0–992.9, “effects of heat and light,”† or External Cause of Injury (E-code) of E900.0, “excessive heat due to weather conditions.” Records with an E-code of E900.1 (i.e., “due to man-made conditions”), and records of patients whose disposition was “death” (n = 75) were excluded. De-identified electronic death certificate data were obtained from the NYC Department of Health and Mental Hygiene Office of Vital Statistics for the years 2000–2011 for NYC residents and homeless persons. Hyperthermia deaths were defined as cases having *International Classification of Diseases, 10th Revision,* (ICD-10) codes X30, “exposure to excessive natural heat” or T67, “heatstroke and sunstroke” listed as causes of death anywhere in the record, for deaths occurring in the months of May–September. Records having a man-made cause of heat exposure (W92) were excluded.

Counts and rates of heat illness ED visits, admissions, and deaths were summarized by age, sex, neighborhood poverty, and place of illness onset (Table 1). Average annual rates were estimated using 2005 population estimates produced by the NYC Department of Health and Mental Hygiene based on the U.S. Census Bureau Estimate Program and housing data from the NYC Department of City Planning. Neighborhood poverty was classified as the percentage of individuals, by postal code, below 100% of the federal poverty level, according to the U.S. Census American Community Survey 2007–2011.

A protocol was established after 2006 whereby the Department of Health hyperthermia investigation team is notified by the Office of the Chief Medical Examiner and the Office of Vital Statistics of deaths involving hyperthermia during or as a result of an extreme heat event, defined in NYC as ≥2 days when the heat index, which incorporates both air temperature and relative humidity, reaches at least 95°F (35°C), or at least 1 day when the heat index reaches 100°F (37.8°C). Electronic records were reviewed at the Office of the Chief Medical Examiner after extreme heat events during 2008–2011 that involved five or more deaths to obtain details on medical history and postmortem height and weight measurements for 48 decedents and information about the home environment for 41 decedents overcome by heat in their own residence.

Diabetes and obesity prevalences were compared with citywide data from the 2010 NYC Community Health Survey.§ Air conditioning prevalence was compared with the 2007 NYC Community Health Survey, and other housing characteristics were compared with the Census Bureau’s Housing and Vacancy Survey for NYC. Confidence intervals were calculated using exact binomial methods for deaths. Statistical software was used for survey data to account for weighting, and normal approximation methods were used for 2010 decennial U.S. Census data.

## Death Certificates and Hospital Data

During 2000–2011, an annual average of 447 NYC residents were treated and released from an ED for heat illness, another 152 were hospitalized, and another 13 persons died. Of the 154 total deaths that occurred over the 12-year period, 70 (45%) died as a result of two severe heat waves in 2006 and 2011. The 2006 heat wave lasted for 10 days, with maximum heat indices above 90°F (32.2°C), three of which exceeded 100°F (37.8°C), and the 2011 heat wave lasted 4 days, with a peak heat index above 110°F (43.3°C). Rates of heat illness and death increased with age, were typically higher among males than females for those aged <65 years, and increased with neighborhood poverty (Table 1).¶ Approximately 3% of hospitalized patients and 5% of decedents were homeless.

Comorbid conditions recorded for hospitalized patients and contributing causes of death from death certificates included cardiovascular disease (64% and 55%, respectively), diabetes (23% and 13%), substance abuse (14% and 11%), and other mental health conditions (24% and 11%). Respiratory conditions were common among hospitalized patients (22%) but not often indicated as contributing causes of death (4%) (Table 1).

## Medical Examiner Case Review

After heat waves during the summers of 2008–2011, the Department of Health reviewed medical examiner records for 48 hyperthermia deaths. The records noted evidence of cardiovascular disease for 36 decedents (75%), acute or chronic substance abuse for 14 (29%), and a history of schizophrenia or schizoaffective disorder for five (10%). Three (6%) of the decedents had cerebral palsy, including one child. Based on postmortem height and weight, 10 (48%) decedents aged 18–64 years with known body mass index were classified as obese, compared with 16% of NYC adults of the same ages in 2010 (p<0.05). The prevalence of diabetes (15%) was not significantly different from the citywide estimate of 9% (Table 2).

Of the 48 decedents, 41 (85%) were overcome by heat in their own home. Of 26 deaths with information available on the presence or absence of air conditioning, 23 (88%) did not have any air conditioner, and the remaining three (12%) had an air conditioner that was broken or not in use. By comparison, 13% of NYC adults reported living in residences without air conditioning in 2007 (Table 3). The proportion of decedents who lived alone (18%) was not significantly different from the percentage of NYC adults who lived alone (14%). Compared with all city residents, a lower proportion of decedents lived in buildings with elevators (23% versus 35%) or in public housing (2% versus 6%), but these differences were not statistically significant (Table 3).

### Editorial Note

During 2000–2011, approximately 600 cases of serious illness and 13 deaths occurred annually in NYC as a result of heat illness. Although these cases do not capture the full spectrum of the health effects of extreme heat, such as exacerbations of chronic conditions leading to hospital admissions or deaths that are not recognized or coded as heat-related (9), cases of hyperthermia and other forms of heat illness can be directly identified, counted, and investigated to better understand risk factors and potential gaps in public health communications and interventions.

Older adults continue to have the highest rates of heat illness and death. However, persons of all ages are at risk, especially those with underlying physical or mental health conditions and those taking medications that can impair thermoregulation (4). The association of obesity with heat-related death in NYC is consistent with biologic evidence that adiposity increases vulnerability to heat exhaustion (4,5).

In NYC, the majority of hospitalized and fatal cases of heat illness occurred in the home. Among the 26 deaths reviewed that had information about the presence of home air conditioning, none of the decedents had a working air conditioner. Aspects of the urban environment can cause city apartments without air conditioning, in some cases, to reach temperatures more than 18°F (10°C) higher than outdoor temperatures on hot days (6).

Unlike findings from a study of a 1995 Chicago heat wave (7), NYC hyperthermia decedents were not statistically more likely to live in multifamily apartment buildings than the general city population. Also, unlike other previous studies (4), NYC hyperthermia decedents were not more likely to live alone. Hyperthermia can progress rapidly, and many persons might not be aware of the warning signs, including lack of sweating in late-stage illness.

What is already known on this topic?

Heat waves cause more deaths in the United States, on average, than any other type of extreme weather event. Older adults, those with underlying physical or mental health conditions, and those without access to working home air conditioning are most at risk for hyperthermia death.

What is added by this report?

During 2000–2011, approximately 447 heat-related emergency department visits, 152 hospital admissions, and 13 heat-related deaths occurred each year in New York City. Higher rates of heat illness and death were associated with older age and neighborhood poverty; chronic physical and mental health conditions were prevalent comorbidities in decedents. Based on medical examiner records for 48 decedents, 85% were exposed at home and, among records with information regarding the presence of air conditioning, none of the decedents had a working air conditioner. Among decedents aged 18–64 years, 48% were obese and another 29% were overweight. Unlike in some previous studies, decedents in this analysis were not more likely to live in multifamily apartment buildings or to live alone.

What are the implications for public health practice?

Rising summer temperatures from climate change, a growing older adult population, and the increasing prevalence of obesity and chronic disease might increase the number of serious heat illnesses and deaths in New York City. Adaptation efforts in urban settings should focus on neighborhoods with high poverty, promoting greater access to air conditioning, and encouraging members of the public to check on vulnerable family members and contacts.

The findings in this report are subject to at least three limitations. First, cases of heat illness identified in this report might not include heat-associated increases in rates of hospital care for other conditions such as cardiovascular disease, renal disease, and diabetes, and do not reflect heat-associated increases in overall mortality rates from natural causes (4,8). In 2006, 100 excess deaths from natural causes in NYC were attributed to a severe heat wave, based on typical summer mortality rates from a statistical model (9). Second, place of injury is not determined by a standard protocol and often is recorded as unspecified or unknown. Similarly, data on air conditioning was available only for a small number of decedents. Finally, the prevalence of obesity in the general NYC population was based on self-reported height and weight, which tends to underestimate obesity.

Although heat waves typically cause less mortality today, compared with years past (10), reductions in summer heat illness and mortality over the past century in NYC might be threatened by rising temperatures, a growing older adult population, and the increasing prevalence of obesity and chronic disease. This investigation highlights risks to vulnerable persons living in NYC homes without air conditioning. Before and during heat waves, outreach to seniors and those with chronic physical and mental health conditions, as well as their caregivers, should emphasize protective measures to avoid heat illness. Susceptible persons should be encouraged to stay hydrated and use air conditioning, if available, during periods of extreme heat. For those without air conditioning who are able to leave their homes, cooling centers and other air-conditioned public places can provide respite during heat waves. Pools, cool showers, or baths also can provide some relief. Surveillance for hyperthermia illness and mortality can help identify local patterns of vulnerability to best target heat emergency response activities and prevention efforts.

## Figures and Tables

**TABLE 1:** Number, percentage, and rate of residents treated for heat-related illness, by selected characteristics, type of treatment, and outcome — New York City, 2000–2011*

Characteristic	Emergency department visits, excluding hospital admissions or deaths (2005–2010)			Hospital admissions, excluding deaths (2000–2010)			Deaths (2000–2011)		
		
	No.	(%)	Average annual rate per million	No.	(%)	Average annual rate per million	No.	(%)	Average annual rate per million
**Total**	**2,680**	**(100)**	**54.9**	**1,675**	**(100)**	**18.7**	**154**	**(100)**	**1.6**
**Female age group (yrs)**									
0–4	21	(1)	12.1	8	(<1)	2.5	0	—	—
5–14	146	(5)	48.2	11	(1)	2.0	1	(1)	0.2
15–34	435	(16)	61.3	35	(2)	2.7	5	(3)	0.4
35–64	414	(15)	41.0	164	(10)	8.9	21	(14)	1.0
65–84	208	(8)	69.3	361	(22)	65.6	26	(17)	4.3
≥85	63	(2)	99.2	206	(12)	177.0	14	(9)	11.0
**Male age group (yrs)**									
0–4	40	(1)	22.0	13	(1)	3.9	2	(1)	0.5
5–14	133	(5)	42.1	14	(1)	2.4	0	(0)	0.0
15–34	519	(19)	75.3	114	(7)	9.0	7	(5)	0.5
35–64	563	(21)	61.9	389	(23)	23.3	53	(34)	2.9
65–84	120	(4)	59.2	272	(16)	73.2	21	(14)	5.2
≥85	18	(1)	65.9	88	(5)	175.8	4	(3)	7.3
**Neighborhood poverty level** †									
Low (<10%)	368	(14)	36.7	233	(14)	12.7	24	(16)	1.2
Medium (10% to <20%)	947	(35)	52.4	614	(38)	18.5	51	(35)	1.4
High (20% to <30%)	647	(24)	55.2	408	(25)	19.0	36	(24)	1.5
Very high (≥30%)	707	(26)	76.5	357	(22)	21.1	36	(24)	1.9
Other/Unknown§	11	—	—	63	—	—	7	—	—
**Homeless** ¶	<6	NA	NA	58	(3)	NA	7	(5)	NA
**Comorbidities**									
Cardiovascular	233	(9)	NA	1,069	(64)	NA	84	(55)	NA
Diabetes	106	(4)	NA	387	(23)	NA	20	(13)	NA
Respiratory	102	(4)	NA	373	(22)	NA	6	(4)	NA
Substance use/dependency	44	(2)	NA	240	(14)	NA	17	(11)	NA
Mental illness	63	(2)	NA	402	(24)	NA	17	(11)	NA
**Place of heat illness**									
Residence	270	(15)	NA	636	(48)	NA	37	(80)	NA
Street	237	(14)	NA	229	(17)	NA	4	(9)	NA
Industrial place or worksite	126	(7)	NA	34	(3)	NA	1	(2)	NA
Other	978	(56)	NA	363	(27)	NA	4	(9)	NA
Unspecified/Unknown§	930	—	—	353	—	—	108	—	—

**Abbreviation:** NA = not available.

* Hospital outpatient data available only for 2005–2010, inpatient through 2010. Data restricted to events in months of May–September.

† Neighborhood poverty level was based on postal code and defined as the percentage of residents with incomes below 100% of the federal poverty level, according to the American Community Survey 2007–2011. Rates were based on 2010 census data.

§ Excluded from the denominator used to calculate percentages.

¶ Based on homeless indicator in hospital data and residence unknown in death certificates.

**TABLE 2:** Number and percentage of heat stroke decedents (n = 48), by selected medical characteristics — New York City, 2008–2011

Medical characteristic	Heat stroke decedents		

	No.	(%)	(95% CI)
Evidence of cardiovascular disease	36	(75)	(60–86)
Evidence of alcohol or substance abuse	14	(29)	(17–44)
History of diabetes	7	(15)	(6–28)
History of schizophrenia/schizo-affective disorder	5	(10)	(3–23)
Cerebral palsy	3	(6)	(1–17)
**Known body mass index among those aged 18–64 yrs (n = 21)**			
Normal/Underweight	5	(24)	(8–47)
Overweight	6	(29)	(11–52)
Obese	10	(48)	(26–70)

**Abbreviation:** CI = confidence interval.

**TABLE 3:** Number and percentage of heat stroke decedents with onset at home (n = 41), by selected housing characteristics,* compared with percentage of city residents overall — New York City, 2008–2011

Housing characteristic	Heat stroke decedents			Residents overall		p-value
	
	No.	(%)	(95% CI)	(%)	(95% CI)	
**Air conditioner in the home**						
Present, working, and in use†	0	—	—	(87)	(87–88)	
Not working or not in use	3	(12)	(3–30)			
No	23	(88)	(70–98)	(13)	(12–13)	<0.001
Unknown§	15	—	—			
**Lived alone**						
Yes	7	(18)	(6–31)	(14)	(14–14)	
No	31	(82)	(66–92)	(86)	(86–86)	0.432
Unknown§	3	—	—			
**Building type**						
1 or 2 units	14	(36)	(21–53)	(33)	(33–34)	
≥3 units, walk-up	16	(41)	(26–58)	(32)	(31–33)	
≥3 units, elevator	9	(23)	(11–39)	(35)	(34–36)	0.262
Unknown§	2	—	—			
**No. of floors**						
≤2	16	(39)	(24–56)	(30)	(29–31)	
3–5	12	(29)	(16–46)	(35)	(34–36)	
≥6	13	(32)	(18–48)	(36)	(35–36)	0.395
**Public housing**						
Yes	1	(2)	(1–13)	(6)	(6–6)	
No	40	(98)	(87–99)	(94)	(94–95)	0.337

**Abbreviation:** CI = confidence interval.

* Comparison with percentage of New York City residents overall is from the 2010 U.S. Census for householders living alone; household occupancy type and structure type is from the 2008 New York City Housing and Vacancy Survey. Comparison with citywide air conditioning prevalence is from the 2007 New York City Community Health Survey for residents living in households with or without air conditioning. Additional information available at http://www.nyc.gov/html/goh/html/data/survey.shtml.

† Percentages for residents overall include all homes with air conditioners, whether working or not.

§ Excluded from the denominator.

## Footnotes

* Complete emergency department records became available in 2005.

† Includes heat stroke, sunstroke, heat syncope, heat cramps, heat exhaustion, heat fatigue, and heat edema.

§ Available at http://www.nyc.gov/html/doh/html/data/survey.shtml.

¶ Additional information available at http://www.nyc.gov/health/trackingportal.
